# Reduced Na_v_1.6 Sodium Channel Activity in Mice Increases *In Vivo* Sensitivity to Volatile Anesthetics

**DOI:** 10.1371/journal.pone.0134960

**Published:** 2015-08-07

**Authors:** Dinesh Pal, Julie M. Jones, Stella Wisidagamage, Miriam H. Meisler, George A. Mashour

**Affiliations:** 1 Department of Anesthesiology, University of Michigan, 7433 Medical Science Building 1, 1150, West Medical Center Drive, Ann Arbor, Michigan, United States of America; 2 Center for Consciousness Science, University of Michigan, Ann Arbor, Michigan, United States of America; 3 Department of Human Genetics, University of Michigan, 4808 Medical Science Building 2, 1241 East Catherine Street, Ann Arbor, Michigan, United States of America; 4 Neuroscience Graduate Program, University of Michigan, Ann Arbor, Michigan, United States of America; Massachusetts General Hospital, UNITED STATES

## Abstract

Na_v_1.6 is a major voltage-gated sodium channel in the central and peripheral nervous systems. Within neurons, the channel protein is concentrated at the axon initial segment and nodes of Ranvier, where it functions in initiation and propagation of action potentials. We examined the role of Na_v_1.6 in general anesthesia using two mouse mutants with reduced activity of Na_v_1.6, *Scn8a* ^*medJ/medJ*^ and *Scn8a* ^*9J/9J*^. The mice were exposed to the general anesthetics isoflurane and sevoflurane in step-wise increments; the concentration required to produce loss of righting reflex, a surrogate for anesthetic-induced unconsciousness in rodents, was determined. Mice homozygous for these mutations exhibited increased sensitivity to both isoflurane and sevoflurane. The increased sensitivity was observed during induction of unconsciousness but not during the recovery phase, suggesting that the effect is not attributable to compromised systemic physiology. Electroencephalographic theta power during baseline waking was lower in mutants, suggesting decreased arousal and reduced neuronal excitability. This is the first report linking reduced activity of a specific voltage-gated sodium channel to increased sensitivity to general anesthetics *in vivo*.

## Introduction

Na_v_1.6 is encoded by the gene *Scn8a* and is one of the major voltage-gated sodium channels in the central nervous system [1,2]. The Na_v_1.6 protein is concentrated within neurons at the axonal initial segment, where it regulates the initiation of action potentials [3,4]. The channel is also concentrated at nodes of Ranvier and is present at lower abundance in soma and dendrites [5–7]. Na_v_1.6 is important for generation of persistent and resurgent currents and is expressed throughout the brain, including in the prefrontal cortex [8], basal ganglia [9], hippocampus [3,10], cerebellum [10–12], and brainstem [13].

Complete loss-of-function mutations of *Scn8a* in the mouse are associated with hind limb paralysis and juvenile lethality [10,14]. Two hypomorphic mutants with partial loss of channel activity, *Scn8a* ^*medJ/medJ*^ and *Scn8a* ^*9J/9J*^, have been characterized. *Scn8a* ^*medJ/medJ*^ has a splice site mutation that does not alter the amino acid sequence but reduces the efficiency of splicing and the level of expression of the Na_v_1.6 protein to 5–10% of wildtype levels [15,16]. The *Scn8a* ^*9J*^ mutation is an in-frame deletion of isoleucine residue 1750 in transmembrane segment DIVS6 that results in a profound reduction of channel activity (http://www.informatics.jax.org/allele/MGI:3838627). *Scn8a* ^*medJ/medJ*^ and *Scn8a* ^*9J/9J*^ homozygotes survive to adulthood and exhibit progressive movement disorders including tremor, ataxia, and dystonia. Reduction of Na_v_1.6 expression in *Scn8a* ^*medJ/medJ*^ mice does not result in compensatory up-regulation of the other major sodium channels Na_v_1.1 and Na_v_1.2 [16].

The interactions of anesthetic drugs with specific voltage-gated sodium channels have been assessed using heterologous assays [17]. In the *Xenopus* oocyte expression system, Na_v_1.6, Na_v_1.2, and Na_v_1.4 are inhibited by the volatile anesthetic isoflurane [18]. Sevoflurane, at clinically-relevant concentrations, did not produce a significant inhibition of the transient inward currents of Na_v_1.8, Na_v_1.7, and Na_v_1.4 expressed in the *Xenopus* oocytes [19]. However, clinically-relevant concentrations of sevoflurane and isoflurane were reported to produce a concentration and voltage dependent inhibition of Na_v_1.4 channel expressed in a mammalian cell line [20]. In mammalian cell line, isoflurane reduces the peak sodium current of Na_v_1.2 by a negative shift in the voltage dependence of channel inactivation and a delay in the recovery from inactivation [21]. Inactivation of the bacterial voltage-gated sodium channel by the volatile anesthetics isoflurane and sevoflurane is proposed to occur by a multisite mechanism [22,23].

*In vivo* studies have also implicated sodium channels in the response to anesthesia, but without specifying the channel subtypes involved. Pretreatment with the sodium channel blockers tetrodotoxin or lidocaine reduced the concentrations of isoflurane and sevoflurane required to produce anesthesia [24,25]. Conversely, administration of the sodium channel activator veratridine decreased the sensitivity to isoflurane [25].

In this study, we investigated the specific role of Na_v_1.6 by examining the *in vivo* response to volatile anesthetics in two hypomorphic mutants with reduced channel activity. We report reduced electroencephalographic theta power and increased sensitivity of both mutant mice to isoflurane and sevoflurane, providing evidence for a functional role of Na_v_1.6 in the maintenance of arousal and anesthetic-induced unconsciousness.

## Materials and Methods

### Mice

All experimental procedures were approved by the University of Michigan Committee on Use and Care of Animals (Protocol #PRO00002561) and were in compliance with the Guide for the Care and Use of Laboratory Animals (8^th^ Edition, The National Academies Press, Washington D.C.) and the ARRIVE guidelines (S1 File). *Scn8a* ^*medJ/+*^ mice have been maintained in our laboratory for 20 years [15]. Homozygous *Scn8a* ^*medJ*^ mice (N = 7) and littermate controls (N = 7) were studied on a (C57BL/6J X C3HeB/FeJ)F1 strain background to avoid the juvenile lethality of homozygotes on the C57BL/6J background [16,26]. The *Scn8a* ^*9J*^ mutation arose spontaneously in strain BALB/cJ at the Jackson Laboratory and has been maintained by backcrossing to strain C57BL/6J. Homozygous mutants (N = 7) and littermate controls (N = 7) were generated by intercrossing heterozygous mice. The *Scn8a* ^*9J*^ mice were from backcross generations N6 and N7 to strain C57BL/6J. Mice were maintained specific pathogen free in ventilated cages on a 12h light: 12h dark cycle (lights on at 6:00 am) with *ad libitum* Picolab Laboratory Rodent Diet 5L0D and water on corn cob bedding. Homozygous mutants were supplemented with soft diet gel 76A from ClearH2O. Homozygous *Scn8a* ^*medJ*^ mice were maintained with the addition of pine shavings to aid movement in the cage. Mice were studied between 3–6 months of age, when their body weights were between 20–28 grams. Littermate controls included heterozygous mutant mice and homozygous wildtype mice, which did not differ in their responses and were combined for analysis. Mice of both sexes were included.

### Surgical procedures

Under surgical levels of isoflurane anesthesia, the mice were implanted with stainless steel screw electrodes over frontal cortex (1.5 mm anterior and 2.0 mm lateral to Bregma) and parietal cortex (1.5 mm posterior and 2.0 mm lateral to Bregma) to record electroencephalogram (EEG). In addition, a pair of insulated wires (Cooner Wires, Inc., Chatsworth, CA), exposed at the tips, were positioned bilaterally into the nuchal muscles to record electromyogram (EMG). A screw electrode over the cerebellum served as the reference electrode for the electroencephalographic recordings. All electrodes were mated with a 6-pin pedestal (Plastics One, Inc., Roanoke, VA) and the entire electrode assembly was affixed to the cranium using dental acrylic (Stoelting Co., IL). Rimadyl (5 mg/kg body wt, s.c.) was administered after surgery for analgesia.

### Electrophysiological recordings and power spectrum density analysis

EEG and EMG were recorded using a Grass Model 15LT physiodata amplifier (15A54 Quad) system (Astro-Med, Inc.) interfaced with a BIOPAC MP-150 data acquisition unit and AcqKnowledge (version 4.1.1) software (BIOPAC Systems, Inc.). Bipolar frontal-parietal and parietal-parietal EEG were band pass filtered between 0.1–100Hz and sampled at 250Hz. The EMG was band pass filtered between 1–100Hz and sampled at 250Hz. Monopolar parietal EEG, referenced to the electrode over the cerebellum and band pass filtered between 0.1–300Hz, was sampled at 1kHz and used for the calculation of power spectral density (PSD) in theta band (4–10Hz). An IIR notch filter was applied to remove 60Hz line noise. Absolute PSD between 4–10Hz was calculated with Welch’s PSD estimate method by segmenting the data into eight Hamming windows with 50% overlap, implemented in the MATLAB Signal Processing Toolbox (MathWorks Inc., Natick, MA). Relative power was calculated for each epoch by dividing the mean absolute power for theta frequency band by the total power across the entire frequency range.

### Experimental design

All experiments were conducted between 10:00 am—2:00 pm in a custom built temperature-controlled airtight clear cylindrical chamber (5.67L) that allows simultaneous electrophysiological recordings and behavioral assessment. The mice were provided at least 10–14 days of post-surgical recovery, during which they were conditioned to the EEG recording cable and the testing chamber for 2–4h for two consecutive days. On the day of the experiment, baseline EEG was recorded for 30 minutes while holding the behavioral state constant by keeping the mice awake using gentle tapping on the outside of testing chamber. The testing chamber was maintained at 37° Celsius with 100% oxygen inflow at 8L/min. The EEG recording continued uninterrupted for the entire experimental session. The inflow and outflow anesthetic concentration was monitored using vapor analyzers (Datex Medical Instrumentation, Inc., Tewksbury, MA). After 30 minutes of baseline wake EEG recording, anesthetic exposure (isoflurane or sevoflurane) started and mice were allowed to equilibrate to each anesthetic level for 15 minutes, following which the anesthetic concentration was increased in 0.1% increments. At the end of 14 minutes of anesthetic exposure, the chamber was turned in order to flip the mouse on its back and assess the loss of righting reflex (LORR), a surrogate for anesthetic-induced unconsciousness in rodents [27–34]. The ataxia in homozygous mutant mice presented a potential confound in the determination of the unconscious anesthetized state based solely on LORR, which depends on motor activity. Therefore, in addition to the behavioral measurement of LORR, we used EEG recordings to assess brain state transitions. The appearance of high-voltage/low-frequency EEG, a defining neurophysiological feature of non-rapid eye movement sleep and anesthetic-induced unconsciousness [31,35–38], was used in conjunction with the onset of LORR as a secondary confirmation. The mice were deemed to have lost consciousness if righting reflex was suppressed for at least 60 seconds and the EEG transitioned from a low-voltage/high-frequency to high-voltage/low-frequency pattern. After achieving anesthetic-induced unconsciousness (LORR), the anesthetic concentration was increased by one more level for 15 minutes, following which the anesthetic exposure was stopped and time to return of righting reflex (RORR), a surrogate for return of consciousness, was recorded. The inflow of 100% oxygen continued until the mice demonstrated full recovery. We also analyzed changes in EEG theta power, a surrogate index for state of arousal in rodents, to compare the anesthetic depth at the point of LORR between the mutants and littermate controls. EEG theta power has been shown to decrease with cortical depression and increase with cortical activation [32,33], supporting its use as a marker for anesthetic state transitions. EEG theta power in the waking state was also compared between the mutant and littermate controls. Theta power at the point of LORR was computed by PSD analysis of 10 minute EEG period from the epoch corresponding to the anesthetic level at which LORR happened. Similarly, a 10 minute EEG epoch was selected just before the start of anesthetic exposure for the computation of pre-anesthesia baseline waking theta power.

The mutant mice and their respective littermate controls were tested independently for isoflurane and sevoflurane and all mice received both anesthetics in a counterbalanced order with an interval of 4–5 days between experimental sessions. Each experiment was conducted two times to ensure the reproducibility of the data and to reduce the margin of error in the determination of the anesthetic concentration at which LORR and RORR occurs. The starting anesthetic concentration was based on a set of preliminary experiments using *Scn8a* ^*medJ/medJ*^ and *Scn8a* ^*9J/9J*^ mice and littermate controls. Isoflurane exposure started at 0.5% for both *Scn8a* ^*medJ/medJ*^ and *Scn8a* ^*9J/9J*^ mice while the respective littermate controls received a starting concentration of 0.6% and 0.7%, respectively. Sevoflurane exposure started at 1.1% for *Scn8a* ^*medJ/medJ*^ and 1.5% for the littermate control group, and at 0.6% for *Scn8a* ^*9J/9J*^ and 1.5% for the littermate control group.

### Statistical analysis

Statistical analysis was conducted in consultation with the Center for Statistical Consultation and Research at the University of Michigan. The number of animals used was based on a similar study from our laboratory [39] and *a priori* power analysis (nQuery Advisor + nTerim, Statistical Solutions Ltd, MA) to ensure that the study had 80% power at an alpha of 0.05. For each mouse the average dose at which it lost righting reflex over two trials was computed. The proportion of mice that lost the righting reflex at each anesthetic dose was graphed and compared between groups. The LORR data were fit to a sigmoidal dose-response curve with a variable slope. Confidence intervals for the curve were computed by maximum likelihood and the delta method. The best-fit EC_50%_ values—effective concentration at which 50% of the subjects loses the righting reflex—were calculated with 95% confidence intervals. Equality between mutant and littermate control groups was tested using likelihood ratio test. The time to recovery of righting reflex (RORR) for isoflurane and sevoflurane between mutant mice and the respective littermate control groups was statistically compared using an unpaired two-tailed t-test. An unpaired two-tailed t-test was used for the comparison of waking theta power between the mutant mice and the respective littermate control groups. The data on RORR and theta power are reported as mean ± standard deviation (S.D.) with confidence intervals (CI) in parenthesis. Statistical comparisons were performed with Graph Pad Prism 6.05 (Graph Pad Software, Inc., La Jolla, CA) and R 3.2.1 (R Foundation for Statistical Computing, Vienna, Austria).

## Results

### Mice with reduced Na_v_1.6 activity exhibit increased sensitivity to induction of anesthesia

**Fig 1:**
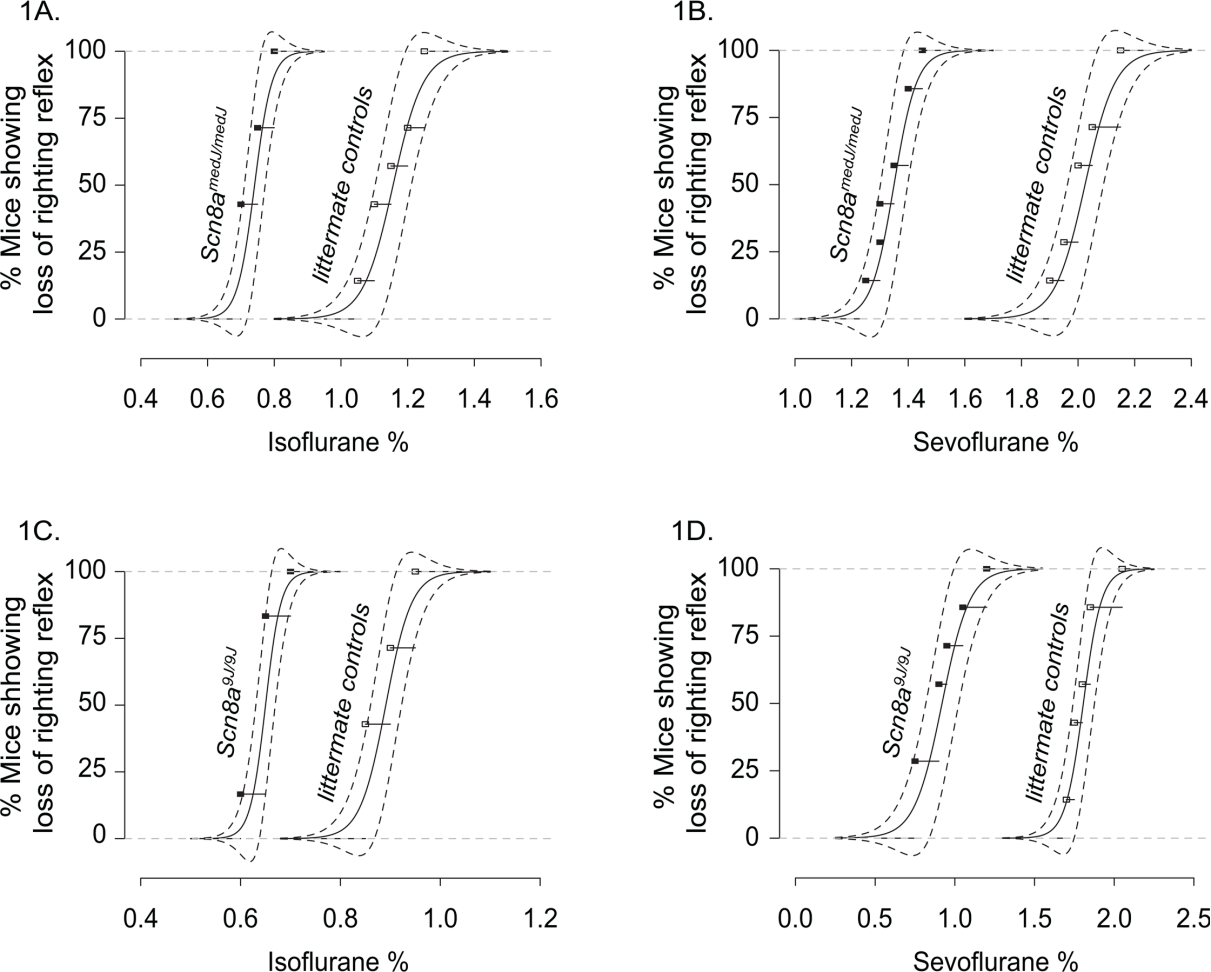
Increased anesthetic sensitivity in mice with *Scn8a* ^*medJ*^ *and Scn8a* ^*9J*^mutations. Best-fit dose response curves along with 95% confidence band (dashed lines) show the behavioral response (loss of righting reflex) of *Scn8a* ^*medJ/medJ*^ **(A and B)** and *Scn8a* ^*9J/9J*^ **(C and D)** mice and their respective littermate control groups to increasing concentration of isoflurane and sevoflurane anesthesia. Seven mice were used for each group. Loss of righting reflex was used as a surrogate for unconsciousness. Filled (mutant mice) and open (littermate controls) squares represents the percentage of mice that lost the righting reflex in response to isoflurane **(A and C)** and sevoflurane **(B and D)**. Data were combined from two trials for each anesthetic and for each mouse. EC_50%_ shows a statistically significant (p<0.0001) leftward shift in the mutant mice, indicating hypersensitivity to anesthesia.

The two mutants tested are associated with different abnormalities of Na_v_1.6 leading to reduced channel activity *in vivo*. *Scn8a* ^*medJ/medJ*^ mice have a reduction in the amount of Na_v_1.6 protein to 10% of wildtype level but no change in the amino acid sequence of the protein. *Scn8a* ^*9J/9J*^ mice have an amino acid deletion in the last transmembrane segment that alters the conformation of the channel pore resulting in loss of channel activity without loss of channel protein. Homozygous *Scn8a* ^*medJ*^ and *Scn8a* ^*9J*^ mice, and their respective littermate controls, were exposed to increasing concentrations of the volatile anesthetics isoflurane and sevoflurane during successive 15 minute periods and the righting reflex was tested at the end of each period. There was a significant shift to the left in the best-fit curves and a decrease in EC_50%_ value for isoflurane and sevoflurane for both *Scn8a* ^*medJ/medJ*^ [Isoflurane: EC_50%_ (95% CI) = 0.74(0.71–0.78) for *Scn8a* ^*medJ/medJ*^ vs 1.2(1.1–1.2) for littermate control group, p<0.0001, chi-square(df) = 38.5(2); Sevoflurane: 1.3(1.3–1.4) for *Scn8a* ^*medJ/medJ*^ vs 2.0(1.9–2.1) for littermate control group, p<0.0001, chi-square(df) = 43.3(2)] **(Fig 1A and 1B)** and *Scn8a* ^*9J/9J*^ [Isoflurane: EC_50%_ (95% CI) = 0.65(0.63–0.67) for *Scn8a* ^*9J/9J*^ vs 0.89(0.86–0.93) for littermate control group, p<0.0001, chi-square(df) = 34.4(2); Sevoflurane: 0.92(0.81–1.0) for *Scn8a* ^*9J/9J*^ vs 1.8(1.7–1.8) for littermate control group, p<0.0001, chi-square(df) = 38.4(2)] mice **(Fig 1C and 1D)**.

As noted in Materials and Methods, real-time neurophysiological features were the criteria used to confirm unconsciousness in addition to the observed LORR. However, in order to demonstrate quantitatively that the more potent anesthetic effects on the righting reflex in *Scn8a* ^*medJ/medJ*^ and *Scn8a* ^9J/9J^ mice were not due to the ataxic phenotype, we compared theta power, an independent measure of electroencephalographic and behavioral arousal, between the mutants and the littermate controls at the point of LORR. The *Scn8a* ^*9J/9J*^ mice and littermate controls showed an equivalent decrease in theta power at LORR produced by isoflurane (64% for *Scn8a* ^*9J/9J*^ vs 58% for littermates) and sevoflurane (12% for *Scn8a* ^*9J/9J*^ vs 17% for littermates), which indicates that the observed sensitivity is due to anesthetic effects on arousal rather than the movement abnormalities. Similarly, the *Scn8a* ^*medJ/medJ*^ mice and littermate controls showed a comparable decrease in theta power produced by isoflurane (46% for *Scn8a* ^*medJ/medJ*^ vs 32% for littermates). The decrease in theta power was 3-fold greater for *Scn8a* ^*medJ/medJ*^ mice compared to littermate controls for sevoflurane-induced LORR (11% for *Scn8a* ^*medJ/medJ*^ vs 35% for littermates).

### Influence of reduced Na_v_1.6 activity on the recovery time from anesthesia

**Fig 2:**
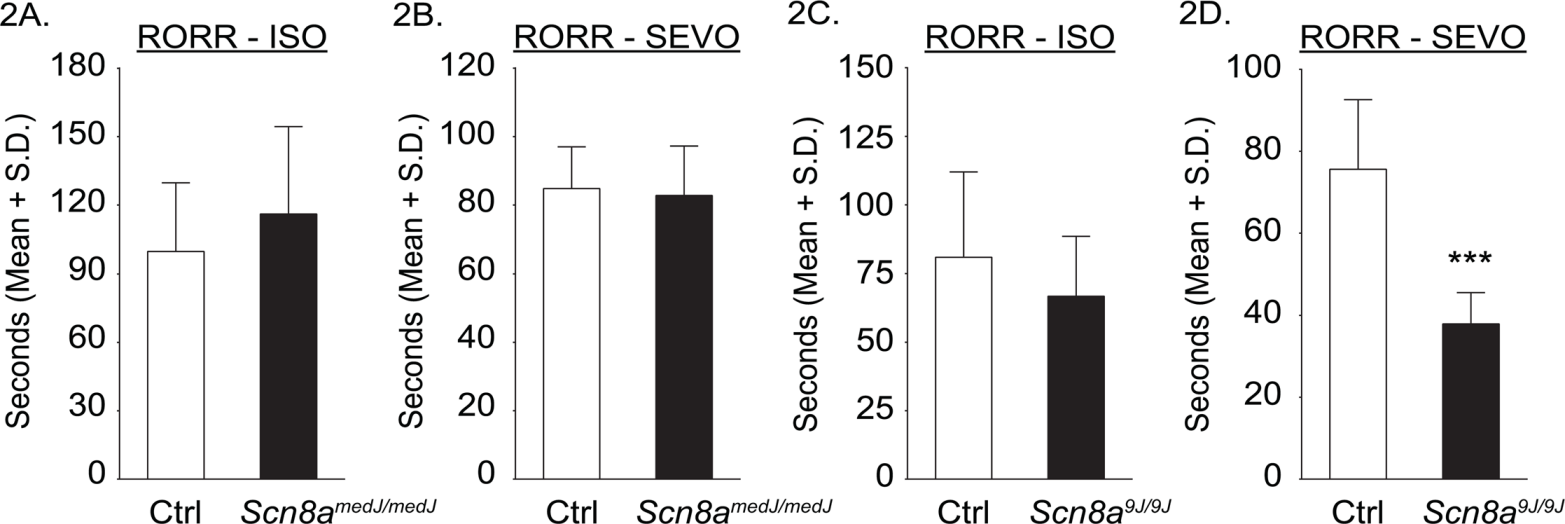
Effect of *Scn8a* ^*medJ*^ *and Scn8a* ^*9J*^mutations on the time to emergence from isoflurane and sevoflurane anesthesia. Compared to the littermate control mice, there was no significant difference in the time to emergence—return of righting reflex (RORR)—from isoflurane anesthesia (ISO) for either *Scn8a* ^*medJ/medJ*^ **(A)** or *Scn8a* ^*9J/9J*^ **(C)** mice, or from sevoflurane anesthesia (SEVO) for *Scn8a* ^*medJ/medJ*^ **(B)** mice. The mice with *Scn8a* ^*9J*^ mutation **(D)** emerged significantly faster from sevoflurane anesthesia. Seven mice were used for each group. Data were combined from two trials for each anesthetic and for each mouse. ***p = 0.0002 compared to littermate control mice (Ctrl). S.D.—standard deviation.

The time required for emergence from anesthesia (recovery of righting reflex, RORR) was not significantly different between *Scn8a* ^*medJ/medJ*^ mice and littermate controls [Isoflurane: Mean ± S.D. (95% CI) = 116s ± 38 (81–152) for *Scn8a* ^*medJ/medJ*^ vs 100s ± 30 (72–127) for littermate control group, t = 0.89, df = 12, p = 0.39; Sevoflurane 83s ± 14 (69–96) for *Scn8a* ^*medJ/medJ*^ vs 85s ± 12 (74–96) for littermate control group, t = 0.28, df = 12, p = 0.78] **(Fig 2A and 2B)**. The time required for emergence from anesthesia for the *Scn8a* ^*9J/9J*^ mice showed an anesthetic dependent effect. There was no significant difference between *Scn8a* ^*9J/9J*^ mice and littermate controls for isoflurane [67s ± 22 (47–87) for *Scn8a* ^*9J/9J*^ vs 81s ± 31 (52–110) for littermate control mice, t = 0.98, df = 12, p = 0.35)], whereas the *Scn8a* ^*9J/9J*^ mice emerged faster from sevoflurane anesthesia [38s ± 7.7 (31–45)] as compared to the control mice [76s ± 17 (60–91)] (t = 5.4, df = 12, p = 0.0002; 95% CI for the difference between means = 22–53) **(Fig 2C and 2D)**.

### Reduced Na_v_1.6 activity depresses EEG theta power during the baseline wake state

**Fig 3:**
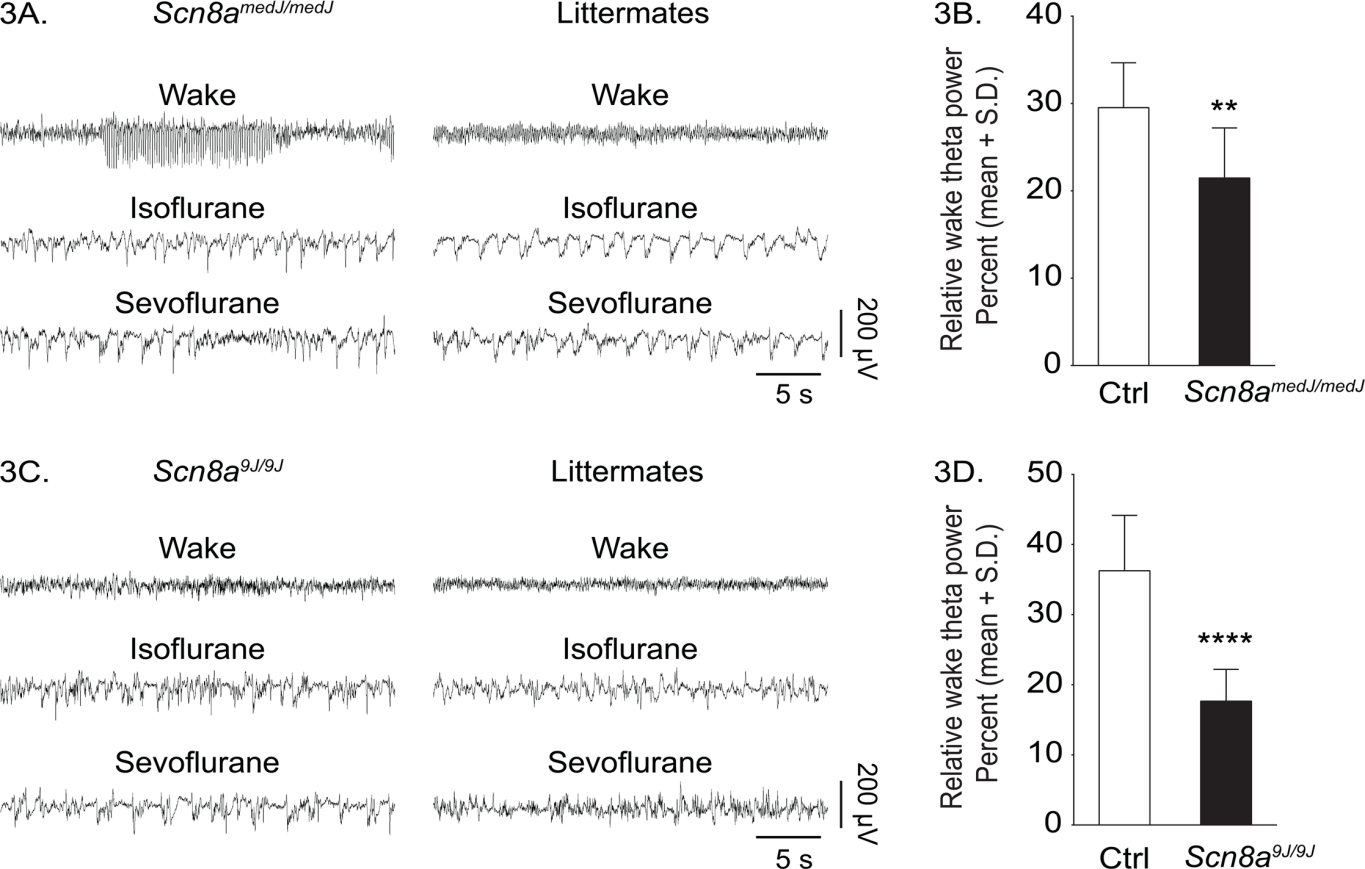
Effect of *Scn8a* ^*medJ*^ *and Scn8a* ^*9J*^mutations on qualitative and quantitative changes in EEG. Representative EEG traces (frontal-parietal derivation) during wake, isoflurane and sevoflurane anesthesia from *Scn8a* ^*medJ/medJ*^mice and the littermate control group **(A)**, and *Scn8a* ^*9J/9J*^ mice and the littermate control group (**C)**. Both *Scn8a* ^*medJ/medJ*^ **(B)** and *Scn8a* ^*9J/9J*^ **(D)** mice show significantly lower theta (4–10Hz) power during waking. **p = 0.001, ****p<0.0001 compared to littermate control group (Ctrl). S.D.—standard deviation.

Since alterations of sleep homeostasis can affect sensitivity to inhaled anesthetics [27,31], we assessed signs of sleep deprivation in the mutant mice during pre-anesthesia baseline wake state. Increased theta power during wake state is a reliable marker for rodents with increased propensity to sleep [40]. Therefore, we analyzed the raw EEG and compared the EEG theta power during pre-anesthesia baseline waking state between the mutant mice and the respective littermate controls. *Scn8a* ^*medJ/medJ*^ mice showed bursts of spike-wave discharge activity during waking, which is indicative of epileptiform EEG, although no overt seizures were observed **(Fig 3A)**. These spike-wave discharges occurred exclusively in association with low muscle tone, as determined by electromyography. The EEG transitioned from high-frequency/low-amplitude pattern during waking to low-frequency/high-amplitude EEG after exposure to anesthesia. In contrast, *Scn8a* ^*9J/9J*^ mice showed a normal wake-related high-frequency/low-amplitude pattern, which transitioned to low-frequency/high-amplitude EEG after exposure to anesthesia **(Fig 3C)**. Spectral analysis showed that the theta power in *Scn8a* ^*medJ/medJ*^ mice was significantly lower than the littermate controls during baseline waking state [Mean ± S.D. (95% CI) = 21% ± 5.7 (18–25) for *Scn8a* ^*medJ/medJ*^ vs 30% ± 5.1 (26–33) for littermate group, t = 3.7, df = 24, p = 0.001; 95% CI for the difference between means = 3.6–13] **(Fig 3B)**. A similar reduction in the theta power was observed for *Scn8a* ^*9J/9J*^ mice [18% ± 4.6 (15–20) for *Scn8a* ^*9J/9J*^ vs 36% ± 7.9 (32–41) for littermate control group, t = 7.6, df = 26, p<0.0001; 95% CI for the difference between means = 14–24] **(Fig 3D).** Reduced theta power in the waking state excludes sleep deprivation as the cause of increased anesthetic sensitivity in the mutant mice.

## Discussion

We demonstrate that decreased Na_v_1.6 activity, either by reduction of Na_v_1.6 protein in *Scn8a* ^*medJ/medJ*^ mice or by an amino acid deletion in *Scn8a* ^*9J/9J*^ mice, increases sensitivity to volatile anesthetics *in vivo*. Based on our finding of reduced theta activity in the waking state, this sensitivity may result from a decrease in neuronal excitability associated with reduced Na_v_1.6 activity (reviewed in [10]). Theta power during the waking state is a surrogate for arousal [32,33] and has been shown to increase with increased cortical excitability [41]. The observed decrease in theta power during waking in the *Scn8a* ^*medJ/medJ*^ and *Scn8a* ^*9J/9J*^ mice is consistent with previous evidence that reduction in Na_v_1.6 activity due to hypomorphic mutations results in decreased excitability in many types of neurons (see Table 1 in [10]).

We also observed frequent bursts of spike-wave discharges in the EEG of *Scn8a* ^*medJ/medJ*^ mice without any evidence of behavioral seizures. These spike-wave discharges disappeared during the isoflurane- or sevoflurane-induced unconscious state. Similar spike-wave discharges associated with immobility and indicative of absence epilepsy have been reported for other *Scn8a* alleles (*Scn8a* ^*8J*^, *Scn8a* ^*med*^, *and Scn8a* ^*med-jo*^) [42,43]. Further studies are needed to confirm the presence of absence epilepsy and the underlying neurobiology for the occurrence of spike-wave discharges in *Scn8a* ^*medJ/medJ*^ mice. These discharges are likely to be influenced by genetic modifiers contributed by strain C3H to the *Scn8a* ^*medJ/medJ*^ F1 background [42,43].

Hysteresis is observed in the general anesthetic concentrations at which an organism loses and regains consciousness, with higher doses required to induce unconsciousness than to maintain it. There is increasing evidence that this asymmetry is not mediated by simple pharmacokinetic mechanisms, but rather by distinct neural systems that control the induction of and emergence from general anesthesia [29,30]. While the Na_v_1.6 mutations increased sensitivity to induction of anesthesia, they did not affect the time to emergence from anesthesia. This asymmetry has two-fold significance. First, it suggests that the effects of the mutation play a specific role in anesthetic state transitions and that the sensitivity does not merely reflect compromised systemic physiology. Physiological compromise would be expected to increase sensitivity during both the induction and recovery phases of anesthesia. Of note, Na_v_1.6 is expressed at a very low level in the cardiovascular system, which further precludes the possibility of a non-specific systemic effect on the arousal state of these mice [44]. Second, it supports the emerging evidence for a distinction between the neurobiology of anesthetic induction and emergence [30,45]. Further work testing the effects of stepwise anesthetic down-titration during emergence will be required to confirm the observation in this model.

There are a number of limitations to this study. First, the mutant mice exhibit ataxia and dystonia which are potential confounds in the determination of anesthetic-induced unconsciousness based on righting reflex. We addressed this problem by conducting simultaneous EEG recording and used the appearance of high-voltage/low-frequency EEG, consistent with an unconscious state [31,35–38], to confirm our assessment of the onset of LORR. Our approach was validated by high reproducibility of LORR data across trials (separated by 4–5 days). Second, although both isoflurane and sevoflurane caused a well-documented reduction in theta activity at the point of LORR [32,33], the extent of theta power depression showed variability across genotypes and anesthetics. Both mutants differed from their genetically matched littermate controls in response to isoflurane and sevoflurane, demonstrating that these differences are not specific to strain background and instead reflect differences in effects of the sodium channel mutations on anesthetic pharmacodynamics. Furthermore, strain background did not influence the identification of unconscious states because determination of anesthetic-induced unconsciousness was made in real time while theta power was calculated off-line after the experiment was completed. Third, we observed a large difference in EC_50%_ for littermate controls on different strain backgrounds. The strain background was C57BL/6J for the *Scn8a* ^*9J*^ mutation and (C57BL/6J X C3H)F1 for the *Scn8a* ^*medJ*^ mutation, which is lethal at 3 weeks on the C57BL/6J background due to a variant in a splicing factor in strain C57BL/6J [14,26,46]. The difference in EC_50%_ between littermate controls from these strains may be related to genetic polymorphisms as previously described for minimum alveolar concentration [47,48]. In our study the genotype of the littermates is identical to the genotype of the mutants. All F1 mice have identical genotypes, with one chromosome inherited from each inbred parent. The C57BL/6J background is likewise identical for the 9J mutants and littermate controls.

## Conclusion

We investigated mice with mutations in *Scn8a* to determine the unique contribution of the sodium channel Na_v_1.6 to the *in vivo* response to general anesthetics. The large effects of these mutations on both anesthetic drug sensitivity and baseline theta power suggest that Na_v_1.6 plays a significant role in maintaining arousal. This effect on drug sensitivity may be relevant to clinical management of patients with early infantile epileptic encephalopathy due to mutations in *SCN8A* [49]. Analysis of mice with reduced activity of Na_v_1.1 and Na_v_1.2 would be valuable for defining the contributions of each of the major neuronal sodium channels to arousal and the response to volatile anesthetics.

## Supporting Information

S1 File ARRIVE guidelines checklist. (PDF)
